# High hydrostatic pressure harnesses the biosynthesis of secondary metabolites via the regulation of polyketide synthesis genes of hadal sediment-derived fungi

**DOI:** 10.3389/fmicb.2023.1207252

**Published:** 2023-06-13

**Authors:** Ludan Deng, Maosheng Zhong, Yongqi Li, Guangzhao Hu, Changhao Zhang, Qingqing Peng, Zhizhen Zhang, Jiasong Fang, Xi Yu

**Affiliations:** ^1^Shanghai Engineering Research Center of Hadal Science and Technology, College of Marine Sciences, Shanghai Ocean University, Shanghai, China; ^2^Ocean College, Zhoushan Campus, Zhejiang University, Zhoushan, China

**Keywords:** piezo-tolerance, Mariana Trench, fungi, polyketide synthesis genes, secondary metabolites

## Abstract

Deep-sea fungi have evolved extreme environmental adaptation and possess huge biosynthetic potential of bioactive compounds. However, not much is known about the biosynthesis and regulation of secondary metabolites of deep-sea fungi under extreme environments. Here, we presented the isolation of 15 individual fungal strains from the sediments of the Mariana Trench, which were identified by internal transcribed spacer (ITS) sequence analysis as belonging to 8 different fungal species. High hydrostatic pressure (HHP) assays were performed to identify the piezo-tolerance of the hadal fungi. Among these fungi, *Aspergillus sydowii* SYX6 was selected as the representative due to the excellent tolerance of HHP and biosynthetic potential of antimicrobial compounds. Vegetative growth and sporulation of *A. sydowii* SYX6 were affected by HHP. Natural product analysis with different pressure conditions was also performed. Based on bioactivity-guided fractionation, diorcinol was purified and characterized as the bioactive compound, showing significant antimicrobial and antitumor activity. The core functional gene associated with the biosynthetic gene cluster (BGC) of diorcinol was identified in *A. sydowii* SYX6, named as *AspksD*. The expression of *AspksD* was apparently regulated by the HHP treatment, correlated with the regulation of diorcinol production. Based on the effect of the HHP tested here, high pressure affected the fungal development and metabolite production, as well as the expression level of biosynthetic genes which revealed the adaptive relationship between the metabolic pathway and the high-pressure environment at the molecular level.

## Introduction

Deep-sea environments below the sea level of 1,000 m, accounts for about 95% of the ocean (Zeng Qi and Fazuo, 2018). The hadal zone, Mariana Trench, localizes the deepest area of the oceans at a depth of 11,000 meters. The conditions in deep-sea environments are extreme, characterized by the absence of sunlight irradiation, predominantly low temperature, and high hydrostatic pressure (HHP) (Wang et al., 2015). Recent studies have shown that the trench harbors abundant sedimentary organic carbon, resulting in unexpectedly high microbial abundance and diversity (Jebbar, 2015; Xu et al., 2018). One of the challenges that extreme microbes meet is the HHP. Several features required for the adaptation to HHP have been clarified in prokaryotes, such as biosynthesis of unsaturated membrane lipids, motilities, and respiratory chain components (Jebbar et al., 2015). There is also evidence that piezo-tolerant and piezophiles microbes have evolved specific metabolic pathways associated with influencing protein in structure and function, increasing the proportion of unsaturated fatty acids in the membrane, etc. (Fang et al., 2010; Mota et al., 2013; Wang et al., 2021; Banerjee et al., 2023).

Fungi that inhabit the sea at a depth of over 1,000 m below the surface are called deep-sea fungi, which are thought to have great potential to produce bioactive metabolites (Zain Ul Arifeen et al., 2019). Deep-sea fungi evolved unique biological metabolic pathways to respond to the extreme ecological environments, including environmental stress (Wang et al., 2020). Fungal natural products from deep sea represent a huge and largely untapped resource of unique chemical structures and novel bioactivities. The first fungus isolated from the deep sea was reported by Roth et al. (1964). Since then, a number of fungal species have been isolated from the deep sea based on culture-dependent methods. Particularly, *Aspergillus* and *Penicillium* belonging to *Ascomycota* are dominant species in deep-sea environments (Wang et al., 2015). Most of these fungi which grow in extreme environments have the potential of producing unique secondary metabolites for defense and communication, some of which hold novel compounds with great potential, thus contributing a strong potential market value for marine biological products (Coleine et al., 2022). Previous studies have shown that 76% of secondary metabolites from marine fungi exhibit bio-activities such as anti-tumor, anti-bacterial, anti-fungal, anti-oxidant, anti-diabetic, anti-inflammatory, anti-cardiovascular, and anti-proliferative, and more than 50% of that have significant cytotoxic activity against human tumor cells (Skropeta, 2008; Shabana et al., 2021). However, the biosynthetic potential has been hampered by the fact that most of the secondary metabolites were extracted in laboratory conditions, leading to the loss of expression of specific functional genes. Few studies have reported the effect of HHP on the secondary metabolites produced by deep-sea fungi.

Diphenyl ether is a common secondary metabolite with a relatively simple chemical structure. It has a wide range of applications and research value in medicine, pesticides, textiles, daily chemical products, and other fields (Feng et al., 2019). Few of these diphenyl ether compounds have been found in nature, but most of them feature good inhibitory activity (Yang et al., 2010; Wu et al., 2016; Liu et al., 2017; 2019). Diorcinol (3, 3-Dihydroxy-5, 5-Dimethyl dimethyl diphenyl ether) and its derivatives are the simplest but widely distributed diphenyl ethers, which are present in the secondary metabolites of a variety of fungi (Yamazaki and Maebayashi, 1982; Oh et al., 1999; Komai et al., 2006; Bunyapaiboonsri et al., 2007; Yang et al., 2010; Zhang et al., 2012; Gao et al., 2013). Diorcinol with a symmetrical chemical structure not only possesses the ability of bacteriostatic activity on *Staphylococcus aureus*, *Mycobacterium tuberculosis*, and *Candida albicans* but also has antitumor activity and hemolytic activity, even as a candidate compound for the treatment of Alzheimer’s disease (Yurchenko et al., 2010; Zhao et al., 2014; Liu et al., 2017). Cheng et al. successfully reconstructed the biosynthetic pathway of diorcinol through a yeast heterologous expression system. The biosynthetic pathway of diorcinol was firstly catalyzed by non-reducing polyketosynthase encoded by gene *AN7909* to generate diorcinolic acid. Then, decarboxylase AN7910 catalyzed diorcinolic acid to decarboxylase to produce C10-deoxy gerflin. Finally, decarboxylase AN7911 catalyzed the decarboxylase of C10-deoxy gerflin to produce diorcinol (Feng et al., 2019). Previous studies showed that diorcinol was produced by a number of fungi, such as *Aspergillus versicolor*, *Aspergillus sydowii*, *Aspergillus tennesseensis*, *Penicillium* sp. and other endophytic fungi (Bunyapaiboonsri et al., 2007; Song et al., 2015; Cimmino et al., 2016; Saetang et al., 2017; Li et al., 2018). However, nothing was known so far about the regulation of biosynthesis of diorcinol derived from deep-sea fungi.

In our work, we reported the isolation and identification of sediment fungi from the Mariana Trench. Among these fungi, the piezo-tolerant fungus-*Aspergillus sydowii* SYX6 was selected as the representative due to its strong antimicrobial activities. The impacts of HHP on its development and secondary metabolites were further studied. The compound diorcinol was purified and characterized from the fungus by large fermentation. The cytotoxic and antimicrobial activities of diorcinol were verified. Furthermore, we identified a core gene associated with diorcinol biosynthesis in *Aspergillus sydowii* SYX6. Based on the determination of gene expression, we found that HHP impacts the biosynthesis of secondary metabolism via the regulation of polyketide synthase gene in deep-sea fungi.

## Materials and methods

### Collection of sediment samples

Sediment samples at about 5,437 m to 10,954 m depth were collected from the Mariana Trench (11° 20’N, 142° 11.5′E) in November 2019. Depths of the collected deep-sea sediment samples A-G were 5,437, 6,477, 7,332, and 10,954 m, respectively (Supplementary Table S1). The sediments were collected from a sealed box corer. The samples were prepared following as described previously (Peng et al., 2021). To prevent contamination, sterilized shovels were used to remove the surface sediments. The core sediment was divided into subsections and directly transferred into sterile plastic bags to avoid any aerial contamination (Damare and Raghukumar, 2008). The bags were stored immediately at 4°C for subsequent experiments.

### Fungal isolation

In our experiment, about 5 g of sediment from the central part of the subsection was scraped off with a sterilized spoon and placed in a sterile centrifuge tube. Forty five milliliters of deep-sea *in-situ* water was added and mixed in the tube. The media used for isolation was the modified Potato Dextrose Agar (PDA, 20% peeled and sliced potato, 1.0% glucose, and 1.5–2% agar, with deep-sea *in-situ* seawater, natural pH). To inhibit bacterial growth, ampicillin (100 μg/mL) was supplemented into the media. One hundred micro liter of the sample was spread on PDA media and the plates were incubated at 28°C for 3–7 days until fungal mycelium was present. The hyphal-tip isolation assay was conducted, in which the tips of hyphae were cut out and placed on a new PDA. It was repeated several times until a pure culture was obtained. Each sediment sample was replicated in triplicate. The phenotype of fungal colonies and pigmentations were observed and recorded every day. Fungal isolates were all cultured on PDA at 28°C and stored at 4°C.

### DNA extraction and sequencing

Fungal isolates were identified by combining morphological observations and internal transcription interval (ITS) sequences. Total DNA was extracted from all fungal strains using the TIAN combi DNA Lyse & Det PCR Kit (TIANGEN BIOTECH (BEIJING) CO., LTD) following the manufacturer’s recommendations for fungi. Nearly full-length ITS sequences were amplified by polymerase chain reaction (PCR) using the primers ITS1 /ITS4 (all primers used in this study were listed in Supplementary Table S2). The PCR assay for ITS sequences included an initial denaturation step at 95°C for 3 min, followed by 35 cycles of 30 s at 95°C, 30 s at 55°C, 1 min at 72°C, and then a final extension step of 5 min at 72°C, before holding at 4°C. The amplified DNA sequences were analyzed by GENEWIZ for sequencing.

### Phylogenetic analyses

For tentative identification, ITS sequences of selected fungal isolates were blasted within NCBI (National Centre for Biotechnology Information^1^) database to determine the nearest relatives in GenBank and aligned using Clustal W in MEGA X. The sequences were trimmed to ensure that all sequences had the same startpoint, following the methods in the previous study (Singh et al., 2011). Phylogenetic trees of ITS sequences were created using the neighbor-joining method with 1,000 replicates were run to calculate bootstrap supports to show the relationship between the isolated strains with the references in the database.

### RNA extraction and quantitative real-time PCR

The cultures of selected fungi were grown in PDB (potato dextrose broth, 20% peeled and sliced potato and 1.0% glucose, with deep-sea *in-situ* seawater, natural pH) for 3 days at 0.1 MPa, 28°C. Vegetative hyphae prior to the onset of sporulation were collected and inoculated in an appropriate amount of PDB in pouches, which were made with sterilized gas-permeable polypropylene sheets and sealed without trapping any air bubbles (Singh et al., 2010). The pouches were suspended in a pressure culture vessel and incubated at 20 MPa and 40 MPa, respectively. The pouches incubated under 0.1 MPa were used as the control. After 21 days, the mycelium treated with different HHP conditions were ground into powder in liquid nitrogen, and then used for total RNA isolation. Total RNA was isolated using RNAiso Plus (TaKaRa, Dalian, China) according to the manufacturer’s instructions. Then cDNA was synthesized using the PrimeScript RT reagent kit with gDNA Eraser (Perfect Real Time) (TaKaRa, Dalian, China). The concentration of RNA was then quantified using a Nano-Drop 2000c spectrophotometer (Thermo Scientific, Wilmington, DE, United States). The qRT-PCR was performed using SYBR Premix Ex Taq II (Tli RNaseH Plus) (TaKaRa, Dalian, China) on the ABI 7500 Real Time PCR System (Thermo-Fisher). The reactions were performed using the following conditions: initial denaturation at 95°C for 30 s, followed by 40 cycles of 95°C for 5 s, and 60°C for 34 s. β-tubulin was chosen as an internal reference gene for normalization and the fold changes were calculated using the formula 2^−ΔΔCT^. The qRT-PCR was performed for triplicate independent experiments.

### High hydrostatic pressures assay on fungal development

Selected sporulating fungi were grown on PDA plates at atmospheric pressure, 28°C. Then the spores were collected by gently scraping the surface of the plates with PDB. The spore suspension was appropriately diluted with PDB containing 2% Tween 80 after counting in a hemocytometer and vortexed for 5 min (Damare et al., 2008). The spore suspension described above was inoculated into a 10 mL syringe and treated with the stimulated HHP. These syringes were suspended in pressure vessel filled with pure water, and pressurized to 20 MPa and 40 MPa, respectively, at room temperature. The same syringes were incubated under 0.1 MPa as the control. Three replicates were maintained for each treatment. After 21 days of incubation, the pressure vessel was depressurized gradually and the syringes were immediately placed on ice to avoid further germination.

To determine the effect of HHP on fungal development, 10 μL of spores’ solution was inoculated on PDA at 28°C under atmospheric pressure. The development of mycelial growth and colony morphology were recorded regularly. The germination activity of fungal spores was determined. The spores and hypha after HHP treatment were stained with calcofluor white (Sigma-Aldrich) as described previously (Damare et al., 2006). The microscopic morphology was observed under a fluorescence microscope (Nikon DS-Ri2).

### Extraction of secondary metabolites

The fungi were cultured on PDA medium at 28°C for 3 days. The mycelium was inoculated in a conical flask containing 50 mL PDB medium and cultured at 28°C for 7 days on a rotating shaker (180 rpm). Spore suspensions were prepared by removing the hyphal fragments through vacuum filtration with 8 layers of sterilized gauze. Then 3 mL of the seed culture was inoculated into a 500 mL conical flask (×30) each containing 40 g rice and 60 mL natural seawater, and incubated stationary for 14 days for large-scale fermentation. The whole culture was extracted twice with an equal volume of ethyl acetate overnight. The solvent was collected and then concentrated at 45°C under reduced pressure using the vacuum rotary evaporator to obtain crude extract.

### Isolation and identification of compounds

The crude extract (4.3 g) was separated into nine fractions (Fr.1-Fr.9) on a silica gel column chromatography using step gradient elution with PET/EtOAc (1/1,1/5, v/v) and then with EtOAc/MeOH (5/1, 1/1, v/v). According to the bioactivity-guided purification strategy, Fr.2 (296 mg) was further purified by Agilent 1,260 HPLC using a column (Agilent Zorbax SB-C_18_, 250 × 9.4 mm, 5 μm) to obtain subfractions Fr.2–1 ~ F2.3 and compound 1 (200 mg, t_R_54 min, MeOH/H_2_O 60/40). The mobile phase was gradient elution with water (solvent A) and methanol (solvent B) and had a flow rate of 1 mL/min. Column thermostat was kept at 40°C and the detector was operated at 210 nm. HPLC and analytic grace solvents used for this study were purchased from Sinopharm Chemical Reagent Co. Ltd. (Shanghai, China).

NMR spectra were collected on a Bruker 500 spectrometer or a JEOL 600 spectrometer using the standard programs. The acquisition data and the chemical shifts were expressed in δ (ppm) relative to DMSO-d6 (δC 39.5 and δH 2.50).

### Antimicrobial activity assay

Kirby-Bauer method was used to test the anti-bacterial and anti-fungal properties of the crude extracts against known pathogens. There were 7 indicator pathogens provided by Shanghai Rainbowfish Company, including *Staphylococcus aureus* ATCC25923, *Enterococcus faecalis* FA2-2, *Escherichia coli* MG1655, *Chromobacterium violaceum* ATCC12472 CV026, *Mycobacterium smegmatis* MC2155, *Salmonella choleraesuis*, and *Candida Albican*. The test sample was quantified at a final concentration of 10 mg/mL by methanol. The indicated pathogens were inoculated in the sterilized LB broth medium at 37°C on a rotary shaker (180 rpm) for 12 h. The culture of indicator pathogens (with an OD of approximately 0.5) was evenly spread 200 μL pathogens solution on the LB agar medium. The circular sterile filter papers (6 mm) were placed on the plate. Six microliter of the test sample was dropped onto filter papers and methanol was used as the negative control. The media was incubated at 37°C for 12 h. The inhibition zones were measured by Image J and compared with the control to determine the antimicrobial activity.

The micro-broth dilution method as described previously was used to further determine the minimum inhibitory concentrations (MIC) (Xin et al., 2012). Two microliters of 2-fold serial dilution of compound (in DMSO) were added to each row on a 96-well microplate containing 100 μL of pathogens suspension in each well. Gentamicin was used as positive control and DMSO was used as a negative control. The 96-well plate was incubated at 37°C aerobically for 24 h.

### Analysis of cytotoxic activity

Human lung cancer NCI-H460, human liver cancer HePG-2, human breast carcinoma MCF-7 and MDA-MB-231 cell lines were obtained from the National Collection of Authenticated Cell Cultures (Shanghai, China). All cells were incubated at 37°C in a humidified incubator with a 5% CO_2_ incubator. Cells after the third generation were used for further experiment.

The assay of 3-(4,5-dimethylthiazole-2-yl)-2,5-dip-henyltetrazolium bromide (MTT) was carried out against the MDA-MB-231, HePG-2, NCI-H460, and MCF7 cell lines. Test samples were dissolved in dimethyl sulfoxide (DMSO) to final concentrations of 200 μM in 96-well plates and each well was placed with 200 μL of cells (3 × 10^3^ per well for cancer cell lines). All the treatments were replicated in triplicate and an equivalent volume of DMSO was used as blank control.

## Results

### Isolation and identification of fungi from hadal sediments

**Figure 1:**
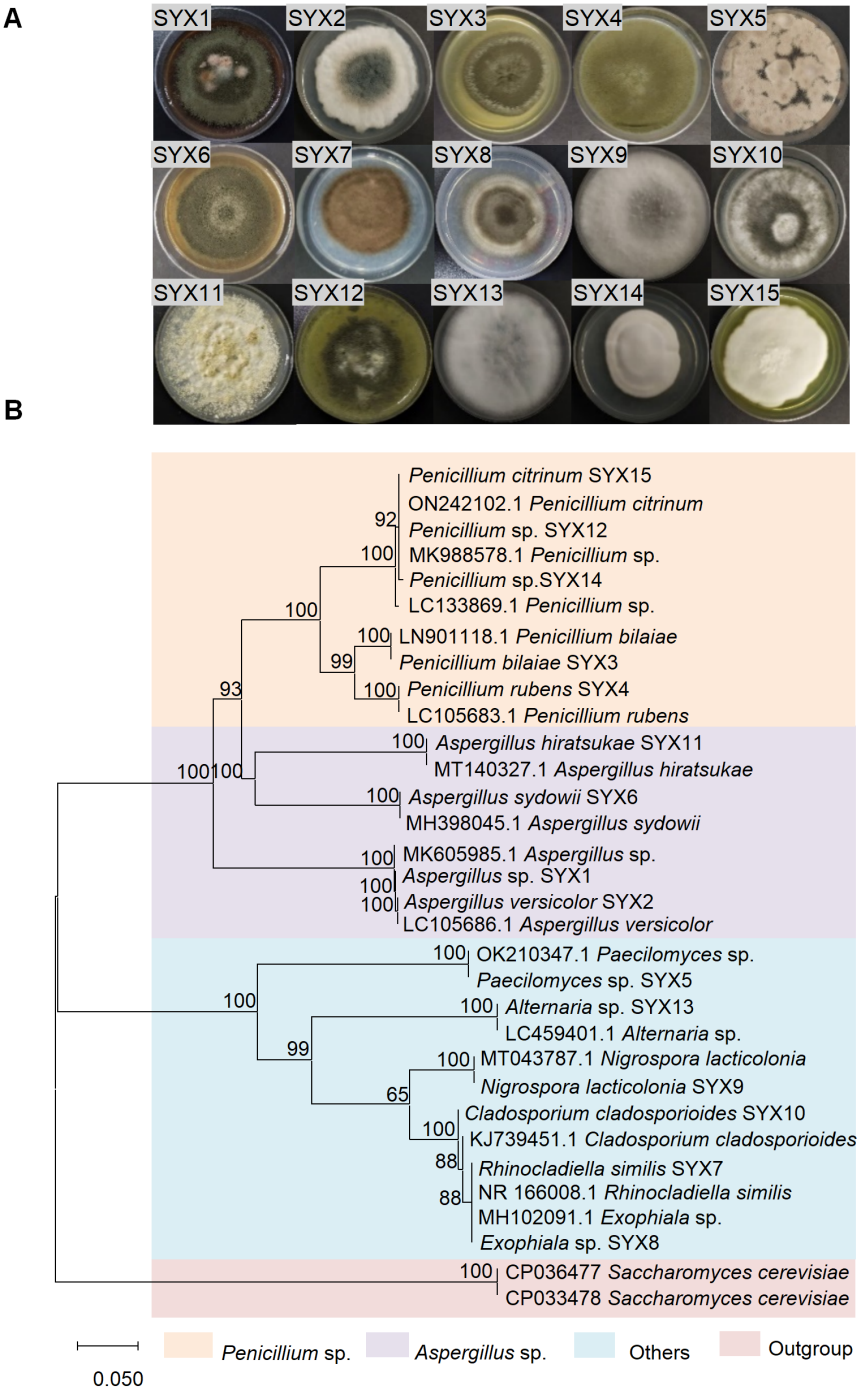
Isolation and identification of sediment-derived fungi from Mariana Trench. **(A)** Colony morphology of 15 fungi cultured on PDA medium for 10 days. **(B)** Phylogenetic tree of ITS sequence (ITS1/ITS4 primers) of 15 deep-sea fungi. The evolutionary history was inferred by using the neighbor-joining method based on the P-distance model and 1,000 bootstrap replicates. The tree is drawn to scale, with branch lengths in the same units as those of evolutionary distances used to infer the phylogenetic tree. Units of the number of base differences per site. Scale 0.050.

A total of 15 fungal strains were isolated from 7 sediment samples of Mariana Trench. Typical morphological taxonomy was recorded after being cultured for 10 days on PDA at 28°C (Figure 1A). Based on morphological identification and ITS sequence analysis (ITS1/ITS4), the isolates were classified as *Aspergillus* sp., *Aspergillus versicolor*, *Penicillium bilaiae*, *Penicillium rubens*, *Paecilomyces* sp., *Aspergillus sydowii*, *Rhinocladiella similis*, *Exophiala* sp., *Nigrospora lacticolonia*, *Cladosporium cladosporioides*, *Aspergillus hiratsukae*, *Penicillium* sp., *Alternaria* sp., *Penicillium* sp., and *Penicillium citrinum* (Figure 1B; Supplementary Table S3). Phylogenetic analysis suggested that *Penicillium* spp. and *Aspergillus* spp. were dominant fungal strains (60% of all isolates) in the Mariana Trench sediments. Our results provided clues about the presence and possibilities of isolating the culturable fungi from the hadal sediments.

### *A. sydowii* SYX6 owns better potential to synthesize bioactive compounds

**Figure 2:**
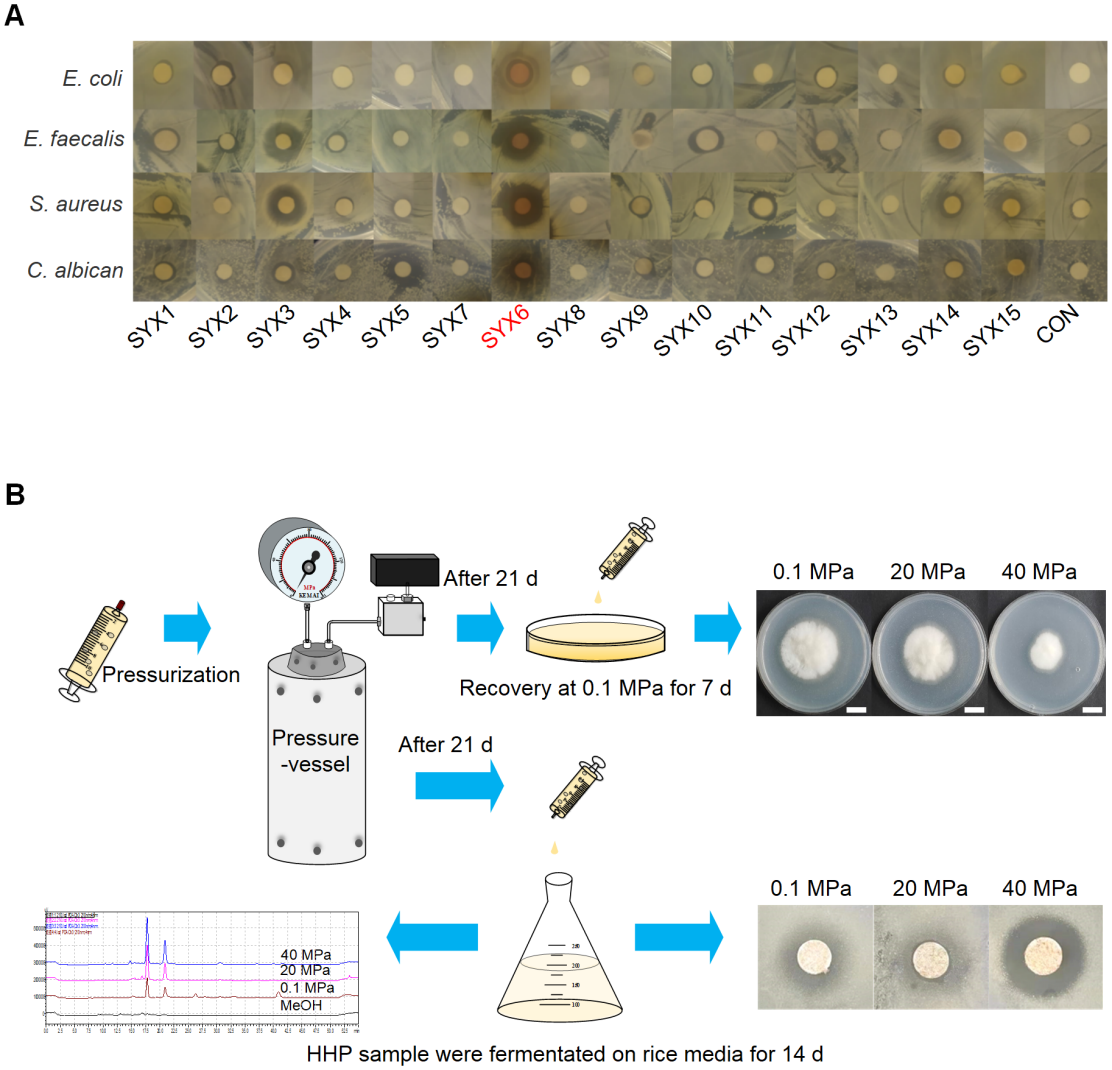
Characterization of deep-sea fungi. **(A)** The antimicrobial activity of secondary metabolites from 15 deep-sea fungi detected by Kirby-Bauer method. *A. sydowii* SYX6 showed the strongest antimicrobial activity. **(B)** Workflow of the high hydrostatic pressure (HHP) treatment assay, including pressurization, recovery culture, determination of fungal development and secondary metabolites production.

To confirm the bioactivities of 15 strains, crude extracts from the isolates were tested for antimicrobial activity toward 7 pathogens with the Kirby-Bauer test. The results showed the certain inhibitory activity of deep-sea fungi against 4 pathogens (*E. coli* MG1655, *E. faecalis* FA2-2, *S. aureus* ATCC25923, and *C. albican*) (Figure 2A). Among them, *P. bilaiae* SYX3 and *A. sydowii* SYX6 showed stronger inhibitory activity with obvious inhibition zones. *P. bilaiae* SYX3 exhibited excellent inhibitory activity against two pathogenic bacteria (*E. faecalis* FA2-2 and *S. aureus* ATCC25923), while *A. sydowii* SYX6 showed significant inhibitory activity against all four pathogens (Figure 2A). The antimicrobial activity of the other strains was weaker or absent. It can be concluded that the genera *Penicillium* and *Aspergillus* from hadal sediment had the most potential for producing bioactive compounds. Among of them, *A. sydowii* SYX6 exhibited the most prominent inhibitory effects.

Preliminary HHP assays on cultivable fungi were performed as described in methods to test the piezo-tolerance of all deep-sea derived isolations (Figure 2B). The results showed that all spore suspensions could re-germinate and develop after incubation at 20 MPa and 40 MPa for 21 days, but spore vitality was decreased and germination was delayed. Through pressure screening, the spore’s viability of *A. sydowii* SYX6 was strongest under the treatment of HHP. Thus, *A. sydowii* SYX6 was selected as the targeted fungus for further investigation because of its significant antimicrobial effects and piezo-tolerance.

### High hydrostatic pressure affects the growth and development of *A. sydowii* SYX6

**Figure 3:**
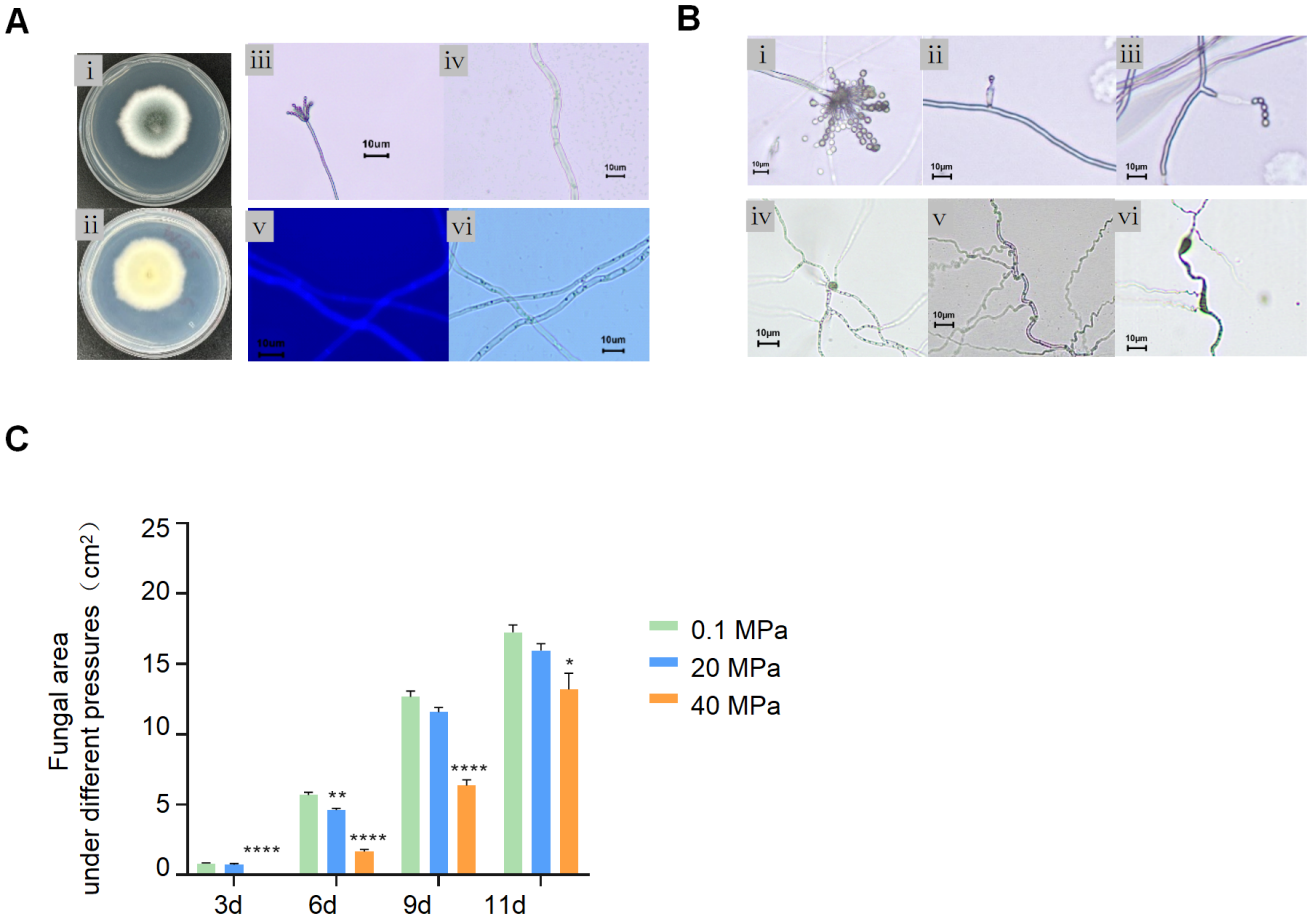
Effect of HHP on *A. sydowii* SYX6. **(A)** Morphological characteristics of *A. sydowii* SYX6. (i, ii) the morphology of colony cultured on PDA for 3 days (front and back). (iii, iv) typical spore and mycelial morphology under microscope. Scale bar: 10 μm. (v, vi) fungal hypha stained with calcofluor white and the same hypha photographed in bright field. Scale bar: 10 μm. **(B)** Morphological characteristic of *A. sydowii* SYX6 under different HHP. (i–iii) Tufted ascospores of 0.1 MPa control group. HHP of 40 MPa affected the development of sporangiophores and inhibited the formation of radiating sporangia clusters. Scale bar: 10 μm. (iv–vi) Hyphae developed in the 0.1 MPa control group. Curled and Swollen hyphae of *A. sydowii* SYX6 under 40 MPa. Scale bar: 10 μm. **(C)** The growth rate of *A. sydowii* SYX6 after treating with different pressure for 3, 6, 9, and 11 days. The unit of the area was cm^2^. Data are the mean ± s.d. *T*-test was used for pairwise comparisons. (*p < 0.05, **p < 0.01, ****p < 0.0001). Plotted with GraphPad Prism 8.

*A. sydowii* SYX6 was a filamentous fungus that produced a blue–green colony and extended 3–4 cm diameter on PDA for 3 days at 28°C. The radiate conidiophores were located at the apex of the sporophores. The septate hyphae were colorless, close-packed, and radiating outward under the observation of microscope. All the hyphae were permeable to the calcofluor white (CFW) (Figure 3A). *A. sydowii* SYX6 belongs to a common genus widely distributed in versatile niches. Our question is whether *A. sydowii* SYX6 evolved a specific adaptative mechanism through suffering a hostile environment in the deep sea. Hence, we further characterized the impact of HHP, one of the key parameters in the deep sea, on the development and growth of *A. sydowii* SYX6.

The spore suspensions were incubated for 21 days under the pressure of 0.1 MPa, 20 MPa, and 40 MPa, respectively. The spore’s germination and phenotype of *A. sydowii* SYX6 under different pressures were determined by microscope. Spores and mycelium germinated normally under atmospheric pressure after treatment with 20 MPa for 21 days. The spores treated under 40 MPa also re-germinated under atmospheric pressure but showed imperfectly formed conidiogenous cells compared to the 0.1 MPa sample (Figure 3B). HHP affected the development of sporangiophores and inhibited the formation of radiating sporangia clusters (Figure 3Bi–iii). The spores were tiny with defective development, showing unusually thick hyphae with swellings and curling (Figure 3Biv–vi). We assumed that even fungal spores have been removed from high pressure, the phenotype was changed as a response to this unfavorable condition, to some certain extent.

Next, we performed phenotype characterization and measured fungal areas cultured under different pressures. The results showed that the fungal colonies extended rapidly on the PDA plates under atmospheric pressure while 40 MPa-treated spores hardly germinated even after 3 days. Although the HHP treated spores were re-germinated on PDA, the growth rate was slower than non-treated spores. While the growth rate of spores under 20 MPa had no distinct difference compared with the control group. We found that 40 MPa significantly delayed the germination of fungal spores (Figure 3C). These results suggested that HHP influenced the germination and vegetative growth of the Mariana Trench sediment derived-fungi.

### Purification and characterization of bioactive compounds via the bioactivity-guided fractionation

The preliminary antimicrobial activity of the crude extract showed the great bioactive potential of *A. sydowii* SYX6. To further characterize the bioactive compounds and understand how high pressure affects the production of secondary metabolites in hadal fungi, an ethyl acetate extract of *A. sydowii* SYX6 was prepared and separated on the basis of its antibiotic properties and spectral methods. In total, 4.3 g of crude extract was obtained from a large-scale fermentation in a rice solid medium. Bioactivity-guided fractionation strategy was performed and the bioactive fraction, compound 1 (200 mg) was isolated. Compound 1 displayed significant antimicrobial activity compared with that of the other compounds from *A. sydowii* SYX6.

**Figure 4:**
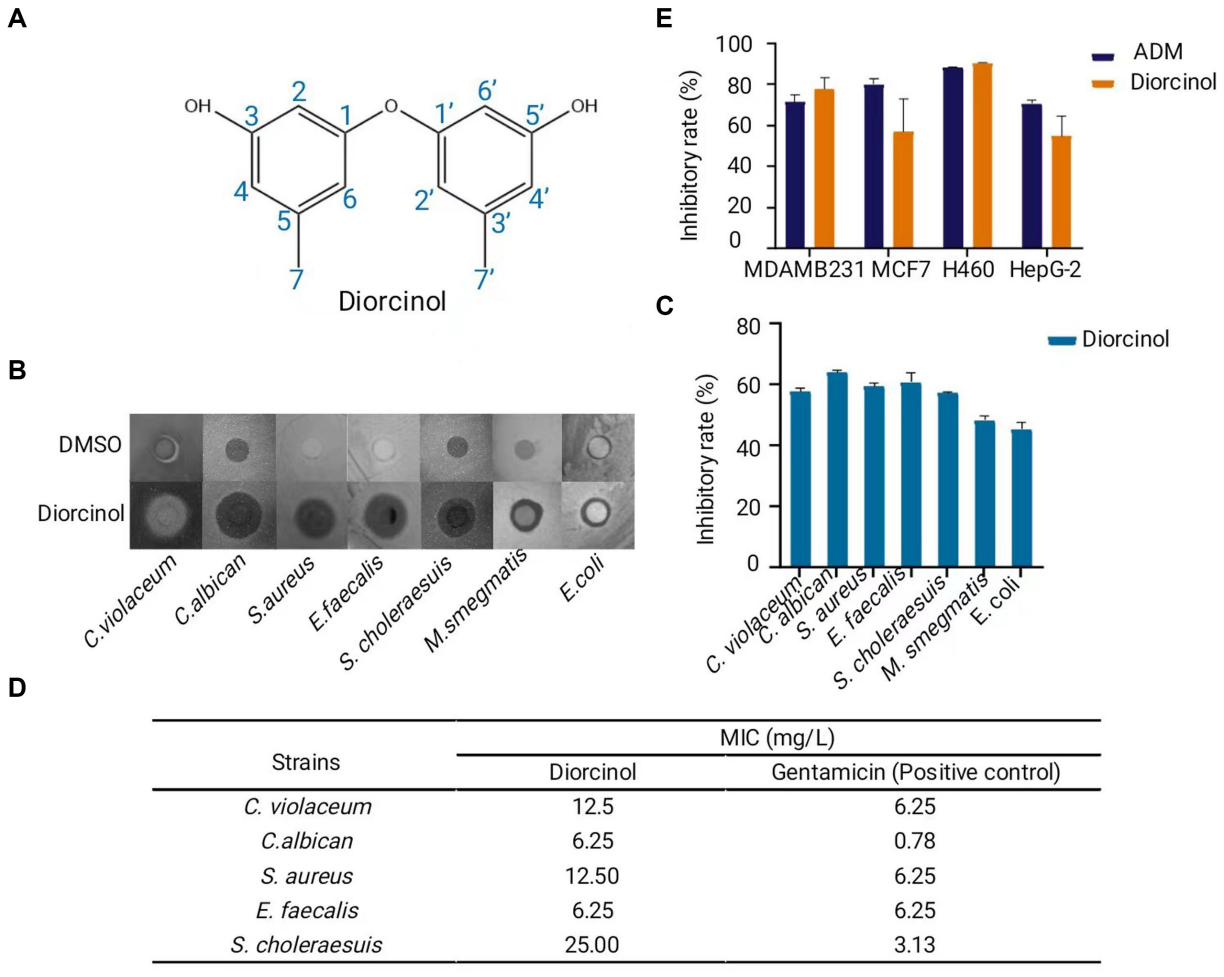
Purification and characterization of the bioactive compound, diorcinol. **(A)** Structure of diorcinol. Plotted with ChemDraw 19. **(B)** Inhibition zone of diorcinol against 7 pathogens indicated by Kirby-Bauer test. DMSO was used as control. **(C)** Inhibition rate of diorcinol against 7 pathogens. The inhibition zones were measured by Image J and compared with the control. Data are the mean ± s.d. The experiments were repeated tree times with similar results. Plotted with GraphPad Prism 8. **(D)** The MIC values of diorcinol against 5 pathogenic strains was determined by micro-broth dilution method. Gentamicin was used as positive control. **(E)** Inhibition rate of diorcinol on different human cancer, including lung cancer NCI-H460, human liver cancer HePG-2, human breast carcinoma MCF-7 and MDA-MB-231 cell lines. Blue squares diorcinol, orange squares adriamycin (ADM). Data are the mean ± s.d. No statistically significant difference was detected compared with the ADM control group (*p* > 0.05). Plotted with GraphPad Prism 8.

Compound 1 was obtained as a yellow oil with a UV absorption at 210 nm. The ^1^H and ^13^C NMR spectrum exhibited signals of two prenyl groups at: ^1^H NMR (600 MHz, DMSO-d_6_ Supplementary Figure S1A) δ: 6.33 (s, 1H), 6.23 (s, 1H), 6.15 (d, J = 1.8 Hz, 1H), 2.18 (s, 3H).^13^C NMR (151 MHz, DMSO-d_6_ Supplementary Figure S1B), δ: 158.43, 157.59, 140.06, 111.15, 110.03, 102.96, 21.11. The ^1^H and ^13^C NMR data were also summarized in Supplementary Table S4. The above data were similar to the spectra data published in the literature (Itabashi et al., 1993; Fremlin et al., 2009; Sanchez et al., 2010; Yurchenko et al., 2010; Huang et al., 2012). Based on the ^1^H and ^13^C NMR spectra and comparison with the data published in previous literature, compound 1 was proven to be identical to diorcinol. Its molecular formula was C_14_H_14_O_3_ (230.09) (Figure 4A).

### Diorcinol shows significant antimicrobial activity and cytotoxicity

To further determine the bioactivity of diorcinol in pharmaceutical potential, human pathogens and tumor cells were chosen to conduct the antibiotic assay. The antimicrobial activity of diorcinol was determined by the Kirby-Bauer test. Diorcinol showed inhibited activity to 7 pathogens (*C. violaceum, C. albican, S. aureu, E. faecalis, S. choleraesuis, M. smegmatis, E. coli*) (Figure 4B), with inhibition rate of 57, 63, 59, 60, 57, 48, and 45% (Figure 4C). Five pathogens with significant inhibition zone were selected as target to determine the MIC value of diorcinol in further. The results of the micro-broth dilution method showed that MIC values of diorcinol against *C. violaceum, C. albican, S. aureus, E. faecalis*, and *S. choleraesuis* were 12.50, 6.25, 12.50, 6.25, and 25.00 mg/L, respectively, whereas the MIC value of positive control were 6.25, 0.78, 6.25, 6.25, and 3.13 mg/L, respectively (Figure 4D).

The MTT assay was used to evaluate the antitumor activities of diorcinol. The results indicate that diorcinol showed a significant cytotoxic effect against human lung cancer NCI-H460, human liver cancer HePG-2, human breast carcinoma MCF-7 and MDA-MB-231 cell lines with the inhibition rate of 90, 55, 57, and 78%, respectively at the concentration of 200 μM. Using the t-test for pairwise comparison, it shows that there was no statistically significant difference (*p* > 0.05) between the inhibition rate of diorcinol and adriamycin (ADM) positive control group (Figure 4E). These findings suggest that the diorcinol, isolated from the culture of deep-sea fungus *A. sydowii* SYX6 has significant inhibitory effects and enormous potential in terms of medicinal value.

### High hydrostatic pressure regulates the mRNA expression level of *AspksD*

The biosynthetic pathway of diorcinol is initiated by the non-reducing polyketosynthase (NRPKS) encoded by the AN7909 gene, which catalyzes the formation of a diphenyl ether-containing polyketide dimer. The structure of diphenyl ether is the core skeleton of natural diorcinol and the formation of ether bonds is the most important step in the biosynthesis pathway of diorcinol (Feng et al., 2019). Genomic mining of a number of fungal strains revealed the widespread distribution of biosynthetic gene clusters (BGCs) containing homologs of AN7909 − AN7910 − AN7911, suggesting that the biosynthesis strategy of diphenyl ether-containing compounds in fungi is conserved, and the AN7909 gene is a critical functional gene in the biosynthesis of diorcinol (Feng et al., 2019).

**Figure 5:**
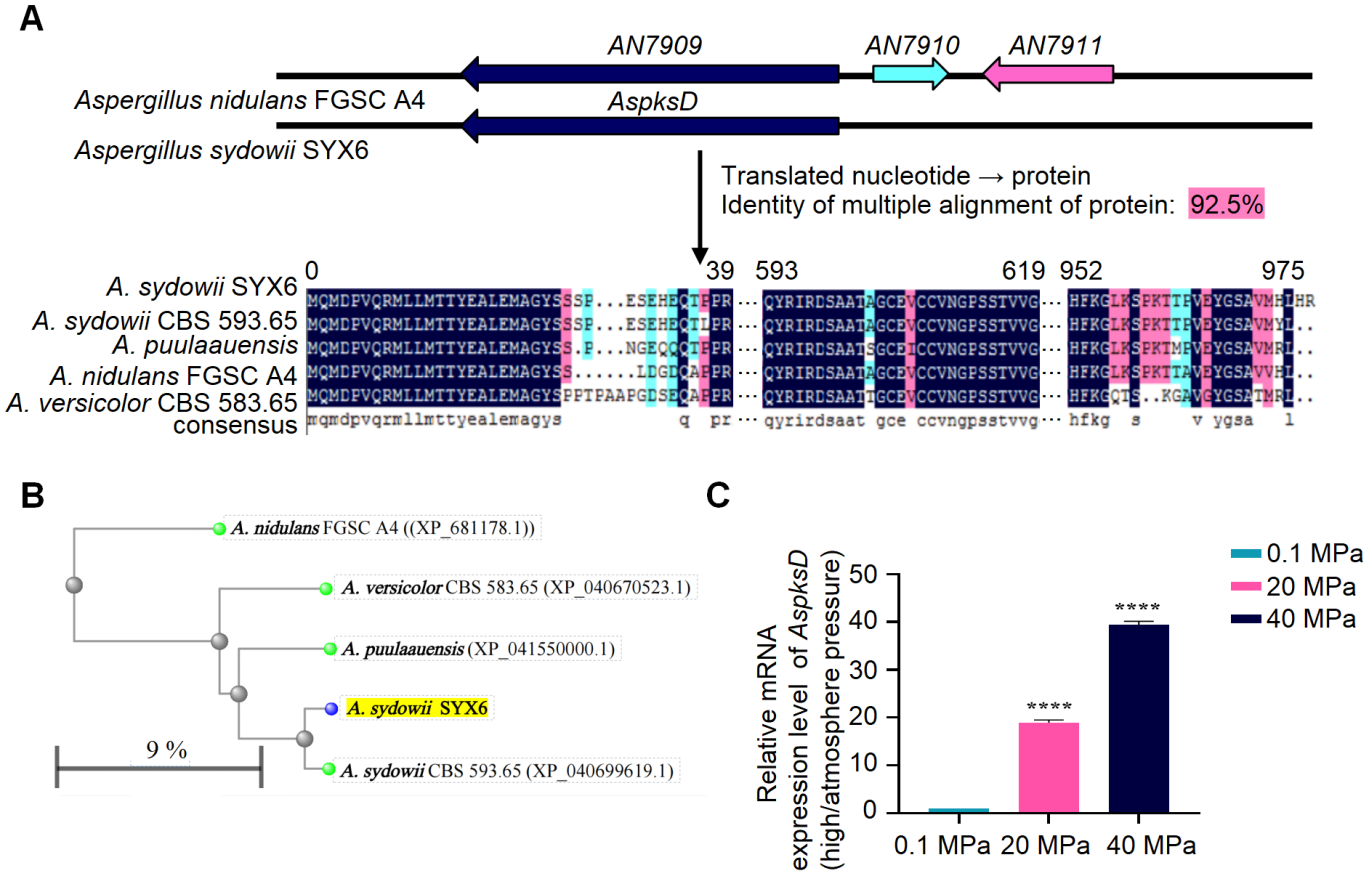
HHP regulates the mRNA expression level of *AspksD*. **(A)** The amplification and sequence alignment of *AspksD*. Amino acid alignment for ASPKSD sequence of *A. sydowii* SYX6 and selected homologues. The homology of multiple alignments in DNAMAN software was 92.5%. **(B)** The distance tree was constructed in NCBI blastp with the neighbor-joining tree method. ASPKSD of *A. sydowii* SYX6 is highlighted. The branch length in the evolutionary tree represents the evolutionary distance between proteins, which is calculated based on the number of replacement sites. In this study, the replacement rate of proteins was estimated to be 9%, indicating that approximately 9% of the amino acid positions in the protein sequences have undergone substitutions. **(C)** Relative mRNA expression level of *AspksD* under different pressure. Error bars represent the standard deviations from three independent experiments. (****p < 0.0001). Data are the mean ± s.d. Plotted with GraphPad Prism 8.

In order to verify the presence of functional NRPKS related genes associated with diorcinol biosynthesis in *A. sydowii* SYX6, the sequence of the *AN7909* homologous gene *OJJ55813* of *A. sydowii* CBS 593.65 was downloaded from NCBI. The primers CF1/SR1 were designed using Primer 5 to locate its key functional region PKSD (Supplementary Table S2). The target gene sequence, named as *AspksD* with a length of 3,059 bp was obtained by amplification (accession number of GenBanK is OQ716674.1). Blastx analysis showed that the amplified fragment had a high homology of up to 97.84% with PKSD related to OJJ55813.1 in *A. sydowii* CBS 593.65, and 83.22% with PKSD related to AN7909.2 in *A. nidulans* FGSC A4. The PKS segments related to AN7909 and its homologous gene clusters of *Aspergillus* were downloaded and, DNAMAN software was used to analyze their kinship with ASPKSD. The results showed that the homology of multiple alignments was as high as 92.50% (Figure 5A). The complete multiple alignment results were also shown in Supplementary Figure S2. A phylogenetic tree was constructed with blastp in NCBI based on the sequence of ASPKSD. ASPKSD and the previously published fungal PKSs originated in the same branch (Figure 5B). This indicates the presence of a key functional gene homologous to the *AN7909* in *A. sydowii* SYX6, laying the foundation for studying the diorcinol biosynthesis under HHP in future research.

The qRT-PCR primers qF2/qR2 were designed based on the *AspksD* fragment in *A. sydowii* SYX6 (Supplementary Table S2). Total RNA was extracted from mycelia cultured under different pressure. The effect of different pressure on the expression level of *AspksD* was detected using qRT-PCR. T-test was used for pairwise comparisons. The results showed that HHP significantly upregulated the expression level of the *AspksD* (****p < 0.0001). Furthermore, the effect of HHP stimulation on the expression of the *AspksD* gradually increased with pressure (Figure 5C). Under 20 MPa and 40 MPa, the expression levels of *AspksD* were 18.9-fold and 40.1-fold higher compared with that of expression under 0.1 MPa, respectively. The expression level of *AspksD* gene was highest under 40 MPa (Figure 5C).

### High hydrostatic pressure influences the secondary metabolites of *A. sydowii* SYX6

**Figure 6:**
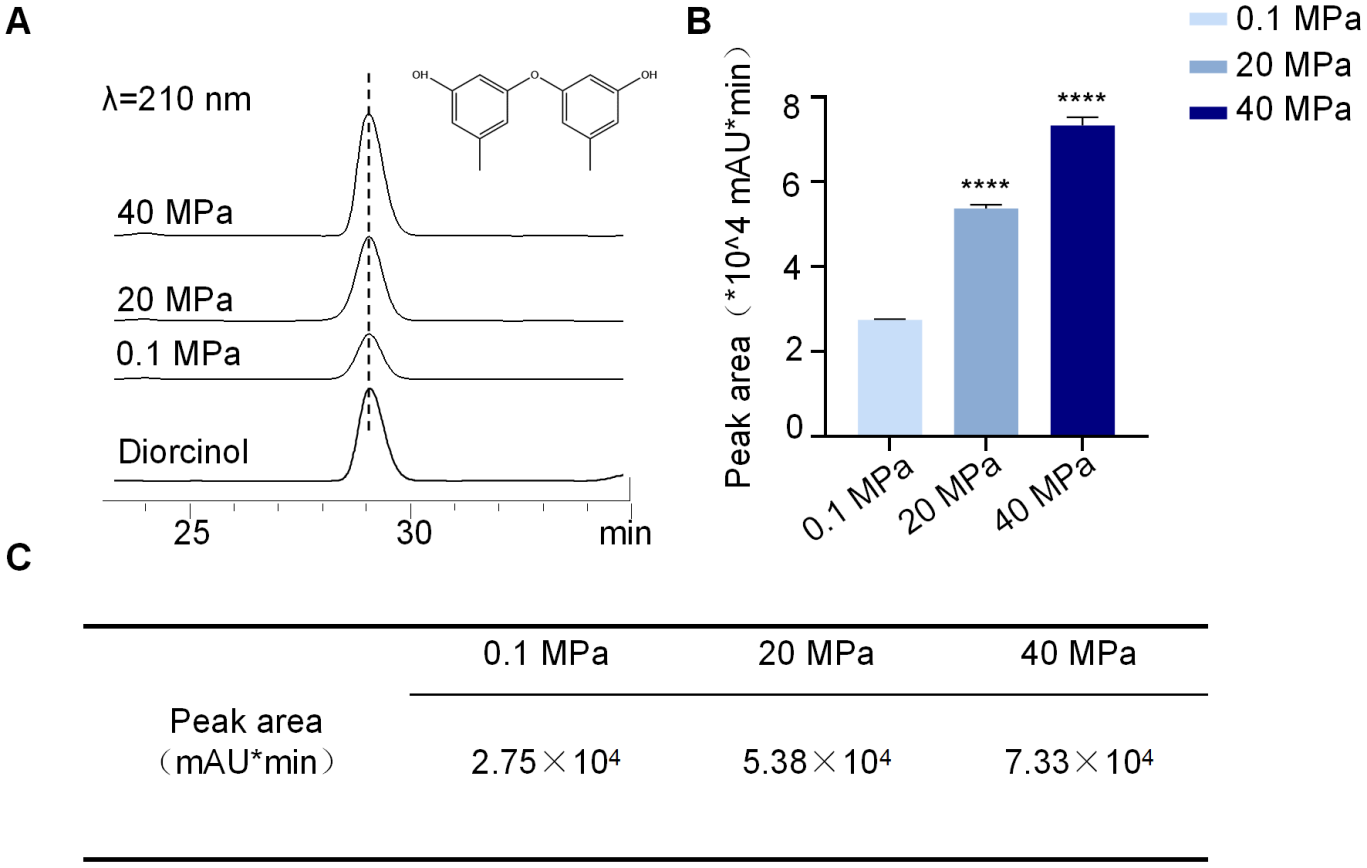
HHP influences the production of diorcinol. **(A)** HPLC peaks of diorcinol under different pressure stimulation, 0.1, 20 and 40 MPa (UV detection at 210 nm). **(B)** Determination of diorcinol peak area under the treatment of 0.1, 20 and 40 MPa. The experiments were repeated tree times with similar results. Statistical significance of the diorcinol production compared with the control group was determined using a two-tailed Student’s *t*-test; the *p* values were as follows: ****p < 0.0001. Plotted with GraphPad Prism 8. **(C)** The production of diorcinol by *A. sydowii* SYX6. The peak areas of diorcinol under 20 MPa and 40 MPa were 1.9 times and 2.7 times of that under 0.1 MPa, respectively. Data are the mean ± s.d. The experiments were repeated tree times with similar results.

To further understand the effect of HHP on secondary metabolites of *A. sydowii* SYX6, spore suspensions treated with various HHP conditions were added to rice media for further fermentation. The production of secondary metabolites of *A. sydowii* SYX6 under 0.1, 20, and 40 MPa were analyzed by HPLC (Supplementary Figure S3). The peak with the same retention time of diorcinol in the crude extract was chosen as the characteristic peak. Peak area was calculated by integrating peak height with retention time and used for quantitative analyzation (Figure 6A). We found that the production of secondary metabolites from *A. sydowii* SYX6 was influenced by the treatment of HHP. The production of diorcinol was expected to increase considerably as the simulated pressure increased (Figure 6B). The peak areas of diorcinol under 20 MPa and 40 MPa were 1.9 times and 2.7 times of that under 0.1 MPa, respectively (Figure 6C). The results indicated that the treatment of HHP influences the production of fungal secondary metabolites.

## Discussion

Previous research on marine fungi mainly focused on sponges and seaweeds, and there have been relatively fewer studies on marine sediment (Debbab et al., 2010; Pang et al., 2016; Zhang et al., 2020). Due to limitations of sampling equipment, research on fungi from hadal sediments is even scarcer (Skropeta, 2008). In recent years, studies of deep-sea fungi using culture and metagenomics methods have provided evidence for the existence of fungi in deep-sea habitats (Feng et al., 2021). Most cultivable and non-cultivable fungi from the deep sea show homology to species described in terrestrial environments (Raghukumar et al., 2010). Representative strains of all genera of fungi have been found in deep-sea habitats worldwide, with up to 80–85% of the known species belonging to the phylum *Ascomycota* according to different kinds of literatures (Zvereva and Borzykh, 2022). In this study, we isolated 15 deep-sea fungi from Mariana Trench sediment samples and identified the bioactive secondary metabolite diorcinol, which expanded the deep-sea fungal library and laid a foundation for the development and utilization of deep-sea microbial resources. All of these belong to known terrestrial genera, with most strains belonging to the *Ascomycota* genera *Penicillium* and *Aspergillus*. This may be due to the fact that both *Penicillium* and *Aspergillus* are halotolerant fungi with prolific growth, making them easy to isolate from various ecological environments. The isolated deep-sea fungi showed a broad range of bioactivities against the indicator strains. The study of fungi derived from deep-sea sediment contributes to discovering new bioactive secondary metabolites for drug development.

Fungi can grow under HHP conditions in the deep sea, although the growth rate may be very slow (Lorenz and Molitoris, 1997; Raghukumar, 2017). HHP creates extreme growth conditions for fungi (Damare et al., 2006). *A. sydowii* strain isolated from 5,904 m of seawater in the Indian Ocean in 2004, was capable of germinating at pressures of 10 MPa, 30 MPa, and 50 MPa (Raghukumar et al., 2004). In our study, under the simulating HHP of a deep-sea habitat, most deep-sea fungi isolates showed tolerance to 20 MPa but were more sensitive to 40 MPa. This was also reflected in their spore vitality. However, we found that *A. sydowii* SYX6 could tolerate and germinate under 40 MPa of pressure, which further proved that *A. sydowii* was a piezo-tolerant fungus. It was reported that spores of terrestrial isolate *A. sydowii* exhibited a complete failure to germinate at 20 MPa (Damare et al., 2006). Our results suggested that *A. sydowii* SYX6 may possess greater tolerance and adaptability to high-pressure environments compared to their terrestrial counterparts. This observation implies a potential specialization and unique tolerance of deep-sea fungi in response to their extreme environmental conditions.

HHP prolonged spore germination time, as observed by Zaunstöck and Molitoris, germination of fungi under deep-sea conditions may experience significant delays (Damare et al., 2008). We believe the delayed germination of spores could be regarded as a protective mechanism, whereby the inhibition of growth enables the fungi to survive and mitigate potential damage in the adverse environment. After the treatment of 40 MPa HHP, the swollen hyphae and abnormal spore germination as observed by us have also been reported by previous studies (Raghukumar and Raghukumar, 1998; Damare et al., 2006). They speculated the spherical cells might be an adaptation by these fungi for pressure tolerance. HHP may reduce the osmosis of solutes inside fungal cells, thereby reducing their intracellular turgor pressure and cell expansion ability (Huang et al., 2014). Hence, the hyphal swelling and curling of *A. sydowii* SYX6 may contribute to unique adaptation to adjust their growth and developmental strategies to respond to their extreme environmental conditions.

The exploration of fungal genomes has considerable prospects for drugs and agricultural chemicals, which has led to in-depth studies of fungal secondary metabolism. In the past few years, bioinformatics algorithms and molecular biology techniques have been combined to greatly promote the identification and research of biosynthetic gene clusters (BGCs) and their natural products (Adpressa et al., 2019; Darma et al., 2019; Li et al., 2019; Lünne et al., 2020; Wolff et al., 2020). Increasing evidences suggest that the regulation of secondary metabolites could be mediated by ecological interactions (Drott et al., 2017). However, there is still a gap in how environmental factors regulate because previous studies have focused more on the endogenous regulation of BGCs. Previous studies using high-throughput sequencing have shown that genes involved in the cell cycle, DNA processing, cell rescue, defense and virulence, and metabolism were highly upregulated under high hydrostatic pressure (Abe, 2007, 2021). In this study, we isolated diorcinol, a known compound with excellent antimicrobial activity and cytotoxic activity, and for the first time, reported the biosynthesis of this NRPKS secondary metabolite in a deep-sea fungus under high pressure. Our data showed that HHP significantly upregulated the expression of the *NRPKS* gene, *AspksD*. When *AspksD* was upregulated, it resulted in a high yield of diorcinol in deep-sea fungi. HHP may promote the expression of BGCs and the production of related secondary metabolites in fungi by activating environmental stress responses. It has been reported that increased pressure at 10°C leads to increased secretion and enhanced activity of proteases in *Aspergillus usstus*, which improves tolerance to lower temperatures when pressure is elevated (Raghukumar and Raghukumar, 1998). Here we demonstrated that the increase in metabolites resulted from the response to pressure activation associated with gene regulation. However, it remains open and needs further investigation to study the physiological functions of natural products derived from deep-sea fungi in a high-pressure environment.

High hydrostatic pressure, as a common characteristic of the deep sea, profoundly affects the metabolic activity of both prokaryotes and eukaryotes, indicating there is a metabolic shift for deep-sea fungi between ambient and high-pressure conditions (Cario et al., 2015; Peng et al., 2021) Therefore, HHP may be a potential factor to increase the yield of deep-sea active secondary metabolites. It was approved that HHP was involved in the regulation of microbial biosynthetic pathways, which may lead to the formation of new products with novel functional properties (Mota et al., 2013). Furthermore, the HHP regulation strategy for *NRPKS* genes may be a new choice for studying the biosynthetic pathways of the latent compound and increasing natural product yield. This is the first report on the adaptation relationship between deep-sea natural diphenyl ether products’ biosynthesis and HHP, which not only provides another idea for the development of deep-sea biological resources, but also opens a new window for using deep-sea fungal diphenyl ether compounds to develop efficient lead drugs. It may also lead to the discovery of other overlooked bioactive compounds in the future.

In summary, we combined HHP with the regulation of key biosynthetic genes in deep-sea fungi for the first time, promoted the expression of functional genes, and achieved an increase in fungal secondary metabolite production, laying a foundation for future pharmacological research. Future research can be expanded from the following aspects: Investigate the fungal diversity and biogeochemical functions in marine sediments of different depths and types, to further reveal the structure and function of marine ecosystems; Screen and identify transcriptional regulatory factors of the *AspksD* gene with its upstream and downstream related genes under HHP; Conduct a broader and more systematic analysis of the changes in secondary metabolites and their biosynthetic genes in other deep-sea fungi under HHP.

### Statistics and reproducibility

Unless otherwise specified, each experiment was independently repeated three times, data were collected from three biological replicates and three technical replicates. The data shown in column graphs represented the mean ± standard deviation (SD) as shown in the figure legends. GraphPad Prism 8 was used for statistical analysis and graph preparation. Chemical structures were prepared using ChemDraw 19.0. More details were given in the figure legend and methods.

## Data availability statement

The datasets presented in this study can be found in online repositories. The names of the repository/repositories and accession number(s) can be found in the article/Supplementary material.

## Author contributions

LD performed most of the experiments and analyzed the results. MZ and YL contributed to the purification and characterization of compounds. GH analyzed the data about the sequence information, CZ and QP participated in the isolation of strains. ZZ analyzed the data about structure characterization. JF reviewed the manuscript. XY designed, coordinated the study, provided all the infrastructure, supervised the project and wrote the manuscript. All authors contributed to the article and approved the submitted version.

## Funding

This work was supported by grants from National Natural Science Foundation of China 42006086, 91951210, and 92251303, Shanghai sailing program 20YF1416900, Science and Technology Commission of Shanghai Municipality STCSM 20050501700.

## Conflict of interest

The authors declare that the research was conducted in the absence of any commercial or financial relationships that could be construed as a potential conflict of interest.

## Publisher’s note

All claims expressed in this article are solely those of the authors and do not necessarily represent those of their affiliated organizations, or those of the publisher, the editors and the reviewers. Any product that may be evaluated in this article, or claim that may be made by its manufacturer, is not guaranteed or endorsed by the publisher.

## Footnotes

^1^ http://www.ncbi.nlm.nih.gov
