# Supplementary material for: High hydrostatic pressure harnesses the biosynthesis of secondary metabolites via the regulation of polyketide synthesis genes of hadal sediment-derived fungi
Source: Front Microbiol. 2023 Jun 13;14:1207252. doi: 10.3389/fmicb.2023.1207252 (PMC10293889; doi:10.3389/fmicb.2023.1207252)
Supplement: Supplementary file 1 [file Data_Sheet_1.PDF]

High hydrostatic pressure harnesses the biosynthesis of secondary metabolites via the regulation of polyketide synthesis genes of hadal sediment-derived fungi

Ludan Deng [1], Maosheng Zhong[1], Yongqi Li[1], Guangzhao Hu[1], Changhao Zhang[1], Qingqing Peng[1], Zhizhen Zhang[2], Jiasong Fang[1], and Xi Yu[1*]

running head: Hadal sediment fungi and bioactive secondary metabolites

Address: [1]Shanghai Engineering Research Center of Hadal Science and Technology, College of Marine Sciences, Shanghai Ocean University, Shanghai, 201306, China

[2]Ocean College, Zhoushan Campus, Zhejiang University, Zhoushan, 316021, China

*Corresponding author: XY (xyu@shou.edu.cn, +8615332036650);

Key words: Piezo-tolerance, Mariana Trench, fungi, polyketide synthesis genes, secondary metabolites

Figure S1

a

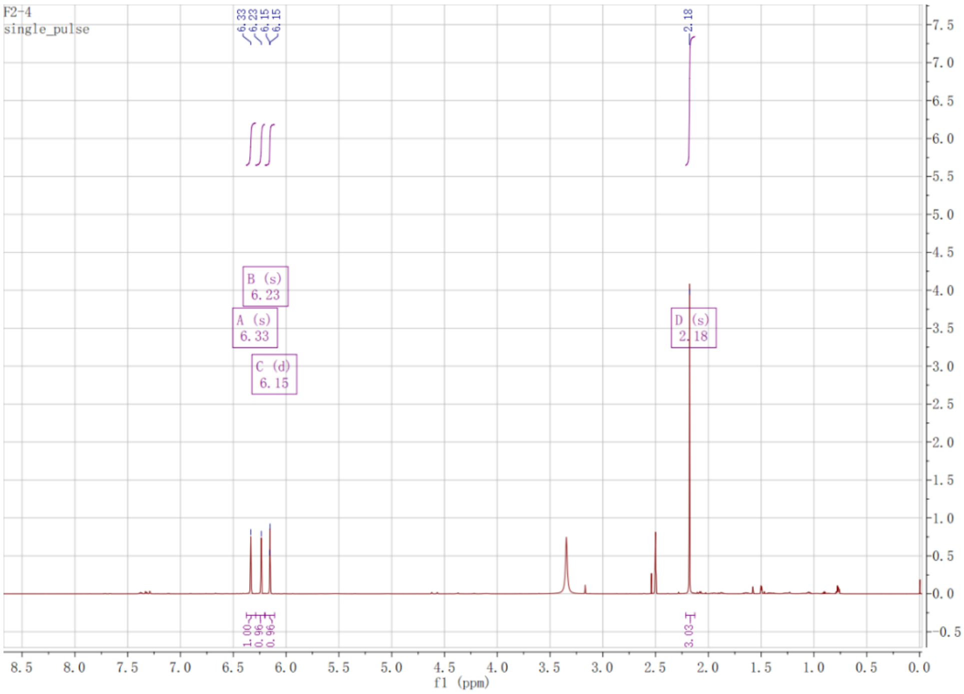

b

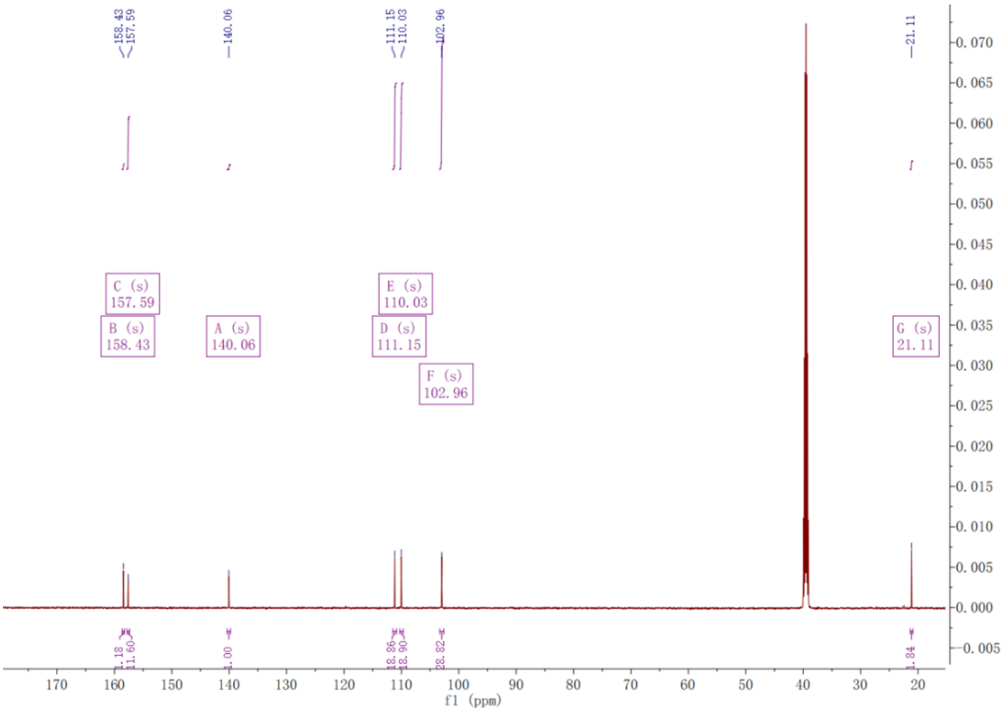

Supplementary Figure S1 Identification of the bioactive compound diorcinol. a, ^{1}H-NMR spectrum of diorcinol in DMSO-d6. **b,** ^{13}C-NMR spectrum of diorcinol in DMSO-d6.

Figure S2

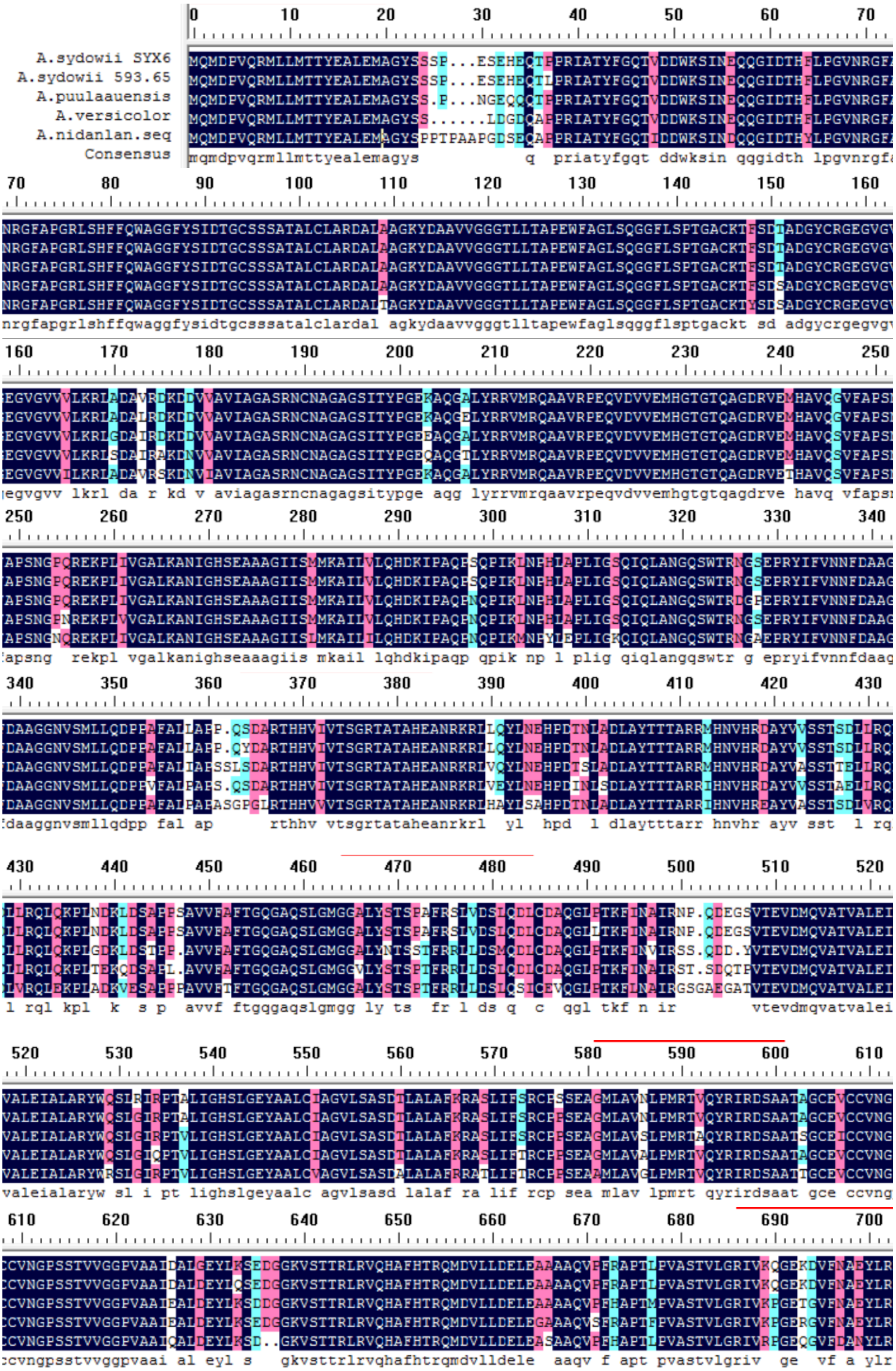
A.sydowii SYX6
A.sydowii 593.65
A.puulaauensis
A.versicolor
A.nidanlan.seq
Consensus

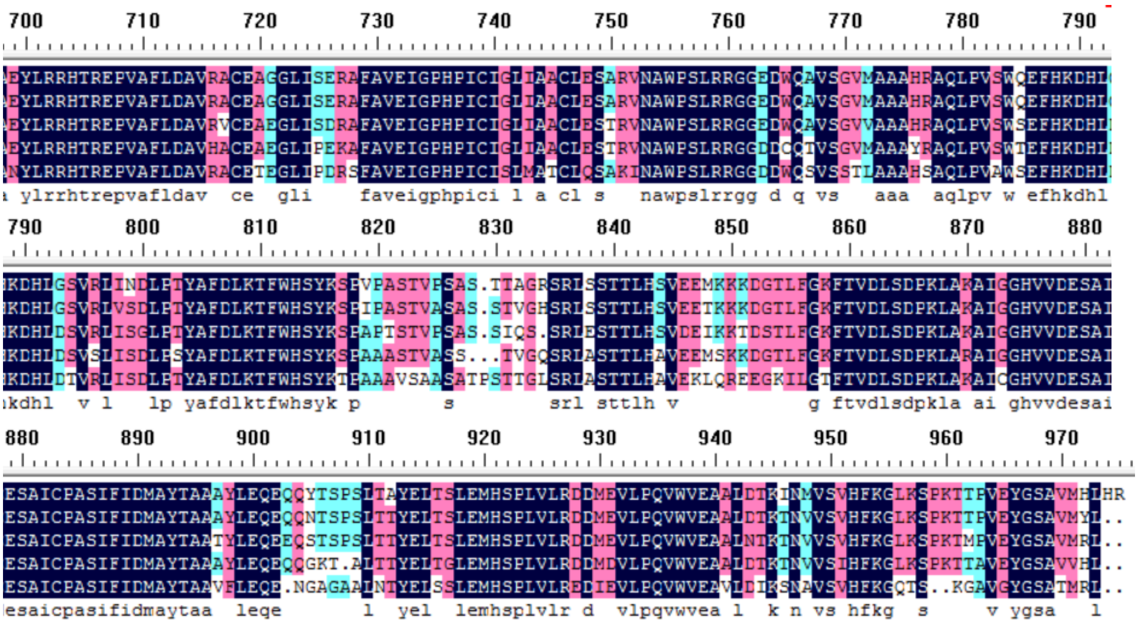

Supplementary Figure S2 Amino acid alignment for ASPKSD sequence of *A. sydowii* SYX6 and selected homologues. The homology of multiple alignments in DNAMAN software was 92.5%.

Figure S3

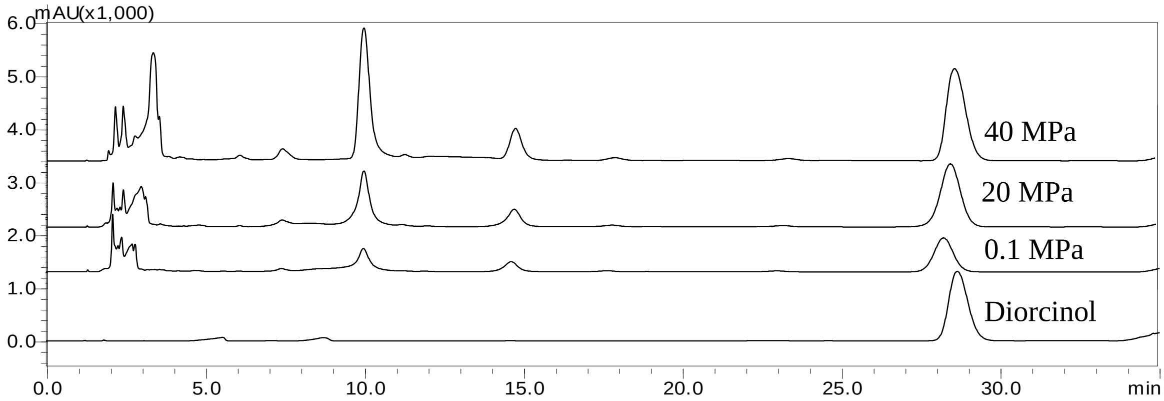

Supplementary Figure S3 Effect of high hydrostatic pressure on secondary metabolism of *A. sydowii* SXY6. High performance liquid chromatography (HPLC) profiling was conducted for secondary metabolites of *A. sydowii* SYX6 after treating with different hydrostatic. (UV detection at 210 nm).

Supplementary Table S1 Information of sediment samples used in this study. The sample was designated A-G. * Approximate depth, as these samples are derived from subsamples of the box core.

Sample	Location	Water depth (m)	Sediment depth(cm)*
A	11.327°N, 142.188°E	10,954	0 - 18
B	10.761°N, 142.274°E	5,437	0 - 10
C	10.761°N, 142.274°E	5,437	10 - 20
D	10.942°N, 141.768°E	7,332	0 - 10
E	10.942°N, 141.768°E	7,332	60 - 70
F	10.813°N, 141.180°E	6,477	0 - 10
G	10.813°N, 141.180°E	6,477	30 - 40

Supplementary Table S2 Primer used in this study.

Primer name	Sequence	Concentration (μM)	Tm (°C)
ITS1	TCCGTAGGTGAACCTGCGG	10	54
ITS4	TCCTCCGCTTATTGATATGC	10	
CF1	ATGTCCGGCCGCTTCCCAGACAG	10	61
SR1	CGCGAATGGTCTCGTATCCA	10	
qF2	GGGGGAATTATACCGGCGAG	10	60
qR2	GGGTCCATTAGACGGTGCAA	10	

Supplementary Table S3 Species information of hadal-derived fungi isolated and cultured in this experiment.

NO.	Sample	Species	Water depth(m)	GenBank accession no.
SYX1	C	*Aspergillus sp.*	5437	OQ712078.1
SYX2	A	*Aspergillus versicolor*	10,954	OQ712077.1
SYX3	A	*Penicillium bilaiae*	10,954	OQ712082.1
SYX4	E	*Penicillium rubens*	7332	OQ712081.1
SYX5	B	*Paecilomyces sp.*	5437	OQ712085.1
SYX6	F	*Aspergillus sydowii*	6447	OQ712076.1
SYX7	D	*Rhinocladiella similis.*	7332	OQ712080.1
SYX8	B	*Exophiala sp.*	5437	OQ712079.1
SYX9	A	*Nigrospora lacticolonia*	10,954	OQ712084.1
SYX10	A	*Cladosporium cladosporioides*	10,954	OQ712078.1
SYX11	C	*Aspergillus hiratsukae*	5437	OQ712074.1
SYX12	C	*Penicillium sp.*	5437	OQ712083.1
SYX13	F	*Alternaria sp.*	6447	OQ712073.1
SYX14	F	*Penicillium sp.*	6447	OQ712086.1
SYX15	F	*Penicillium citrinum*	6447	OQ712072.1

Supplementary Table S4 Identification of the bioactive compound diorcinol. ^{1}H-NMR (600 MHz) and ^{13}C-NMR (150 MHz) spectroscopic data for diorcinol in DMSO-d_6.

**NO.**	**δ_H, (J in Hz)**	**δ_H reference (Sanchez et al., 2010)**	**δ_C**	**δ_C reference (Sanchez et al., 2010)**
1/1’			158.43	159.8
2/6’	6.15 (d，J = 1.8， 1H)	6.19, s	102.96	104.3
3/5’			157.59	159.7
4/2’	6.33，(s，1H)	6.35, s	111.15	112.1
5/3’			140.06	141.8
6/4’	6.23，(s，1H)	6.26, s	110.03	111.9
7/7’	2.18，(s，3H)	2.22, s	21.11	21.7

Sanchez J F, Chiang Y-M, Szewczyk E, Davidson A D, Ahuja M, Elizabeth Oakley C, Woo Bok J, Keller N, Oakley B R, Wang C C C (2010). Molecular genetic analysis of the orsellinic acid/F9775 gene cluster of Aspergillus nidulans. Molecular BioSystems, 6(3): 587-593
